# Supplementary material for: Complex‐centric proteome profiling by SEC‐SWATH‐MS
Source: Mol Syst Biol. 2019 Jan 14;15(1):e8438. doi: 10.15252/msb.20188438 (PMC6346213; doi:10.15252/msb.20188438)
Supplement: Supplementary file 7 — Dataset EV6 [file MSB-15-e8438-s007.zip › feature_plots_bioplex/P51648.pdf]

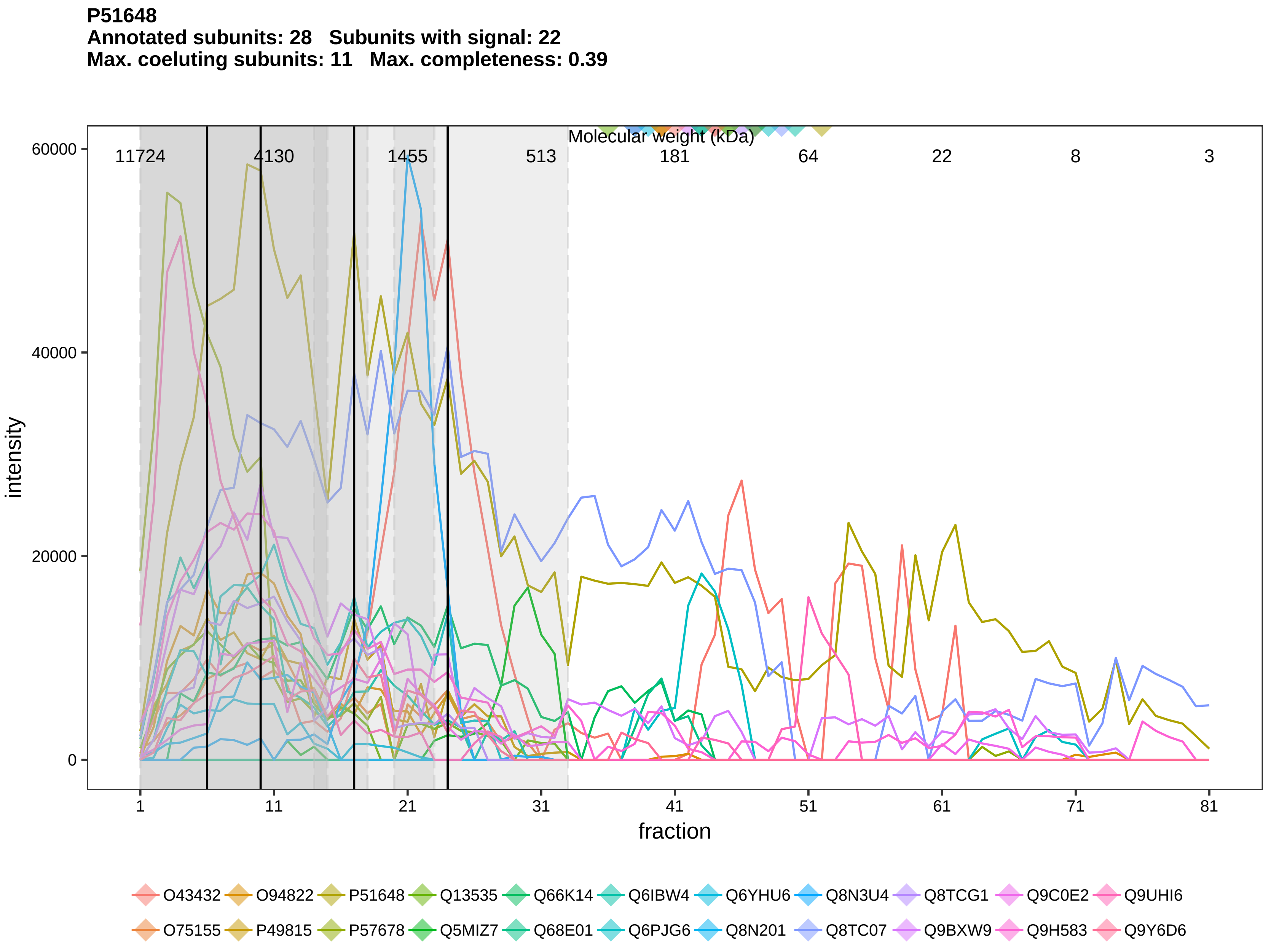
P51648
Annotated subunits: 28 Subunits with signal: 22
Max. coeluting subunits: 11 Max. completeness: 0.39
Molecular weight (kDa)
11724
4130
1455
513
181
64
22
8
3
intensity
60000
40000
20000
0
1
11
21
31
41
51
61
71
81
fraction
O43432
O94822
P51648
Q13535
Q66K14
Q6IBW4
Q6YHU6
Q8N3U4
Q8TCG1
Q9C0E2
Q9UHI6
O75155
P49815
P57678
Q5MIZ7
Q68E01
Q6PJG6
Q8N201
Q8TC07
Q9BXW9
Q9H583
Q9Y6D6
